# Analysis of Surface EMG Signals to Control of a Bionic Hand Prototype with Its Implementation †

**DOI:** 10.3390/s25175335

**Published:** 2025-08-28

**Authors:** Adam Pieprzycki, Daniel Król, Bartosz Srebro, Marcin Skobel

**Affiliations:** Department of Computer Science, University of Applied Sciences in Tarnow, ul. Mickiewicza 8, 33-100 Tarnow, Poland; d_krol@atar.edu.pl (D.K.); b_srebro@atar.edu.pl (B.S.); m_skobel@atar.edu.pl (M.S.)

**Keywords:** EMG, bionic hand, artificial neural network (ANN)

## Abstract

The primary objective of the presented study is to develop a comprehensive system for the acquisition of surface electromyographic (sEMG) data and to perform time–frequency analysis aimed at extracting discriminative features for the classification of hand gestures intended for the control of a simplified bionic hand prosthesis. The proposed system is designed to facilitate precise finger gesture execution in both prosthetic and robotic hand applications. This article outlines the methodology for multi-channel sEMG signal acquisition and processing, as well as the extraction of relevant features for gesture recognition using artificial neural networks (ANNs) and other well-established machine learning (ML) algorithms. Electromyographic signals were acquired using a prototypical LPCXpresso LPC1347 ARM Cortex M3 (NXP, Eindhoven, Holland) development board in conjunction with surface EMG sensors of the Gravity OYMotion SEN0240 type (DFRobot, Shanghai, China). Signal processing and feature extraction were carried out in the MATLAB 2024b environment, utilizing both the Fourier transform and the Hilbert–Huang transform to extract selected time–frequency characteristics of the sEMG signals. An artificial neural network (ANN) was implemented and trained within the same computational framework. The experimental protocol involved 109 healthy volunteers, each performing five predefined gestures of the right hand. The first electrode was positioned on the brachioradialis (BR) muscle, with subsequent channels arranged laterally outward from the perspective of the participant. Comprehensive analyses were conducted in the time domain, frequency domain, and time–frequency domain to evaluate signal properties and identify features relevant to gesture classification. The bionic hand prototype was fabricated using 3D printing technology with a PETG filament (Spectrum, Pęcice, Poland). Actuation of the fingers was achieved using six MG996R servo motors (TowerPro, Shenzhen, China), each with an angular range of $180^{\circ }$, controlled via a PCA9685 driver board (Adafruit, New York, NY, USA) connected to the main control unit.

## 1. Introduction

### 1.1. Surface Electromyography sEMG

**Figure 1:**
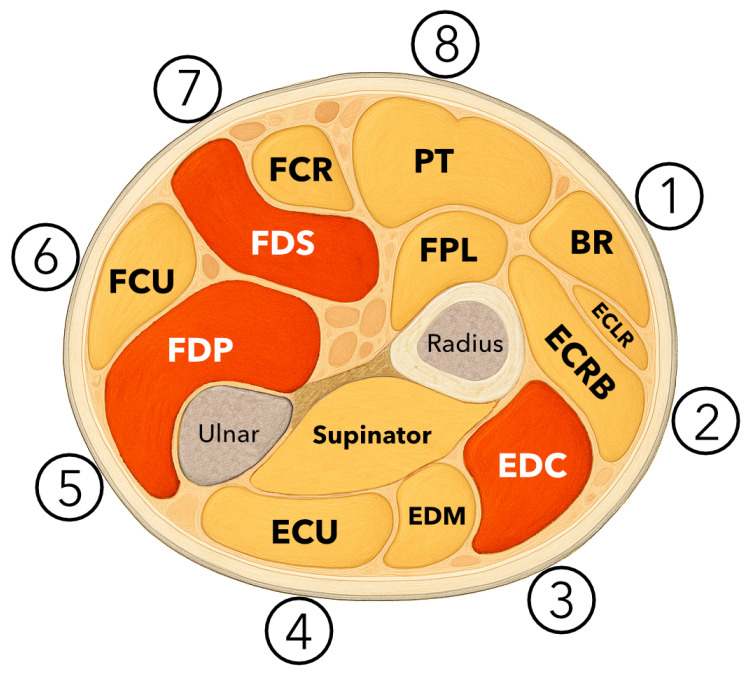
Visualization cross-section forearm with location and numbering of surface EMG electrode.

Electromyographic (EMG) signals are electrophysiological signals generated by muscles in response to stimulation from the nervous system. Among the nineteen muscles located in the forearm, ten are primarily responsible for flexion and extension at the metacarpophalangeal and interphalangeal joints of the fingers and thumb. The most relevant muscles in this context appear to be the flexor digitorum superficialis (FDS), the flexor digitorum profundus (FDP), and the extensor digitorum communis (EDC), as illustrated in Figure 1. EMG signals were acquired in accordance with the SENIAM (Surface Electromyography for the Non-Invasive Assessment of Muscles) guidelines [1].

**Figure 2:**
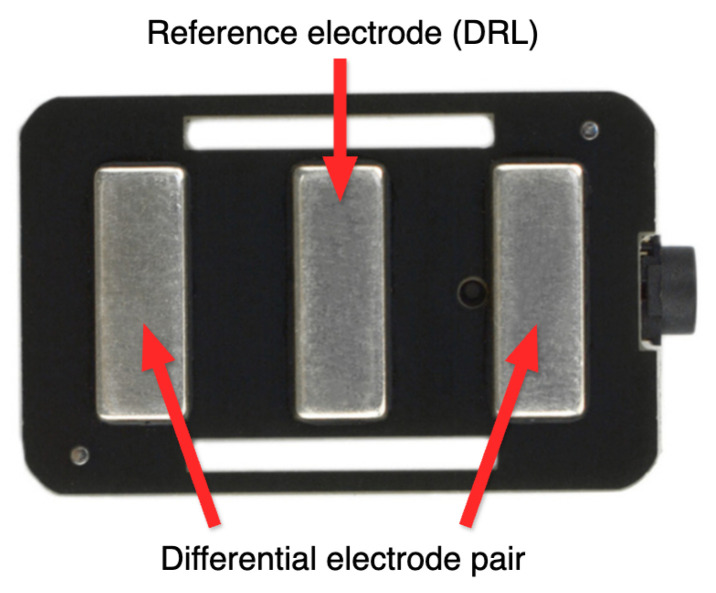
One surface EMG sensor OYMotion SEN0240 (DFRobot, Shanghai, China) with a differential electrode pair and reference electrode DLR (Driven Right Leg).

Electromyographic (EMG) analysis enables the assessment of electrical activity in muscles engaged during different phases of movement. The characteristics of the recorded signal—both in the time and frequency domains—are influenced by several factors, including the number of motor units within the electrode detection range (Figure 2), the type and diameter of muscle fibers, and the frequency of neural excitation that triggers muscle contraction. It must be emphasized that, due to the complexity of neuromuscular physiology, a direct linear relationship cannot be assumed between the amplitude of the EMG signal recorded from a given muscle and the force generated by that muscle.

### 1.2. Principles of Surface Electromyography (sEMG)

Surface EMG (sEMG) is a non-invasive technique that allows for the measurement of muscle activity through electrodes placed on the skin surface. As described by Merletti and Farina [2], sEMG signals are generated by the summation of motor unit action potentials and are influenced by anatomical, physiological, and technical factors. These include inter-electrode distance, electrode size, placement, skin impedance, and crosstalk from nearby muscles. Signal characteristics such as amplitude, frequency content, and duration vary depending on the intensity of contraction and the muscle type [3].

### 1.3. Importance, Applications, and Evolution of sEMG

Since its inception as a needle-based technique, EMG has evolved into a surface-based modality better suited for clinical and engineering applications. While intramuscular EMG offers high spatial resolution, sEMG provides a safer and more comfortable alternative for long-term and mobile measurements [2,4].

sEMG is widely used in various fields, including neurophysiology, biomechanics, rehabilitation, and biomedical engineering. It plays a central role in gesture recognition systems, human–machine interfaces, robotic control, and the development of myoelectric prostheses [5,6]. In clinical settings, sEMG helps evaluate neuromuscular disorders, monitor therapy outcomes, and support biofeedback-based interventions. Despite its advantages, limitations such as electrode placement sensitivity and signal contamination must be carefully considered [4].

### 1.4. Prior Work on Forearm EMG Signal Analysis

The forearm is a common region for sEMG-based control due to the presence of multiple muscles responsible for fine motor control. Previous research, such as the work by Atzori et al. [7], evaluated a wide range of features in the time and frequency domain extracted from multichannel EMG signals of the forearm for the purpose of controlling prosthetic hands. Their study emphasized the importance of robust feature extraction techniques and the impact of factors such as electrode configuration and inter-subject variability on classification performance.

### 1.5. Signal Processing Techniques for sEMG

EMG signal processing typically involves denoising, feature extraction, and classification. Time domain features like root mean square (RMS), zero-crossings, mean absolute value (MAV), and waveform length provide intuitive representations of muscle activity [8]. Frequency domain analysis, especially via Fast Fourier Transform (FFT), offers insight into muscle fatigue and motor unit behavior.

In recent years, time–frequency methods, such as wavelet transforms, have gained popularity due to their ability to analyze non-stationary and dynamic EMG signals. These approaches enable localized tracking of muscle activation patterns and are particularly effective in gesture recognition and motion segmentation [5,8].

### 1.6. Summary and Motivation

**Figure 3:**
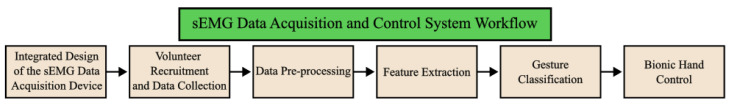
Workflow for sEMG signal acquisition and bionic hand control.

Given the broad applicability and richness of sEMG signals, this study focuses on analyzing forearm sEMG data to support gesture recognition tasks. Understanding the physiological basis of EMG, its historical development, and the standard processing techniques provides a strong foundation for the proposed methodology (Figure 3).

## 2. Materials and Methods

### 2.1. Gesture Signals Collecting—Hardware

Electromyographic signals were acquired using a prototypical LPCXpresso LPC1347 ARM Cortex M3 (NXP, Eindhoven, Holland) development board. The system is based on a 32-bit ARM Cortex-M3 microprocessor operating at a clock frequency of 72 MHz. It features a 12-bit successive approximation register (SAR) analog-to-digital converter (ADC) equipped with an 8-channel input multiplexer and a hardware sequencer for automatic channel switching [9].

Surface electromyographic (sEMG) signals were acquired using Gravity OYMotion SEN0240 (DFRobot, Shanghai, China) sensors (dimensions: 3.5 × 2.2 cm) (Figure 2) connected to dedicated ports of the ADC multiplexer. Each sensor consists of a triple dry electrode module and a signal amplification unit with a fixed gain of 60 dB. The sensor integrates a signal conditioning circuit that includes both amplification and analog filtering. It amplifies low-amplitude sEMG signals within the range of ±1.5 mV by a factor of approximately 1000 and suppresses noise—particularly power line interference—via a differential input and analog filter design. The analog output is centered around a reference voltage of 1.5 V, with a total output voltage range of 0–3.0 V [10].

The use of analog sEMG sensors is non-invasive and user-friendly, making them suitable for applications in human–computer interaction. The dry metal electrodes offer long-term usability and ease of application. Additionally, the sensors feature a differential input with a high common-mode rejection ratio (CMRR), enhancing signal quality in noisy environments.

To enable continuous acquisition, real-time visualization, and multi-channel recording of the EMG signals in WAVE format via a USB 2.0 interface, a dedicated application was developed in C++ using the Qt 5.12.12 framework.

### 2.2. Collecting sEMG Signal Selected Gestures

**Figure 4:**
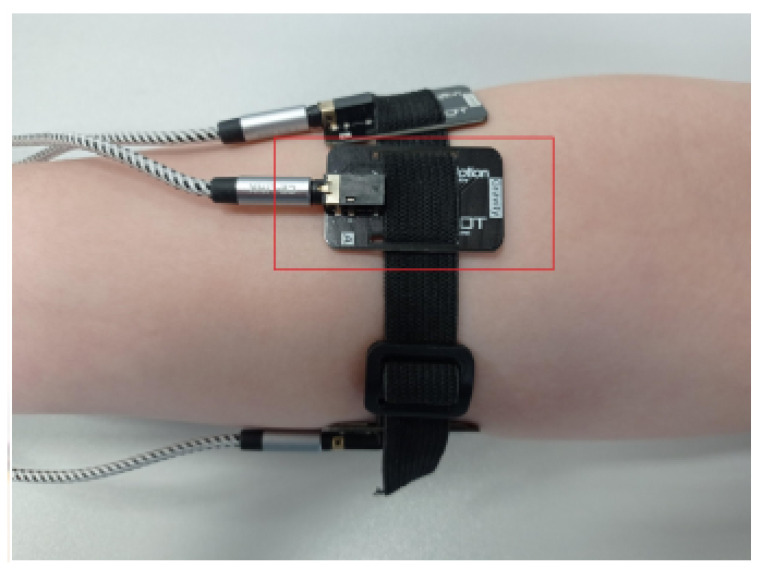
Prototype installation and positioning of the first electrode [11], reproduced with permission from author P. Wawryka [12].

Eight surface electrodes for electromyographic (EMG) signal acquisition were positioned at the mid-forearm level, as illustrated in Figure 4.

**Figure 5:**
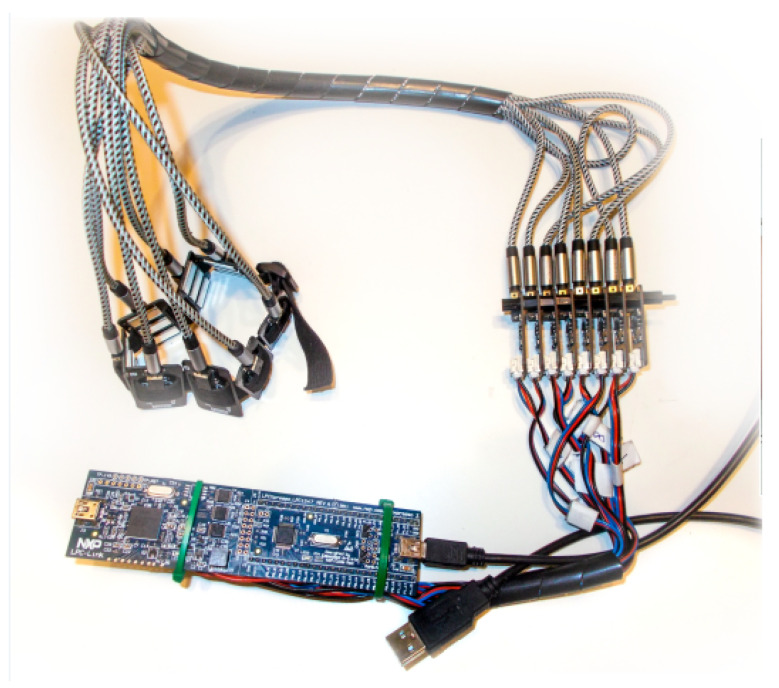
Prototype board and band for the analysis of EMG signals [11], reproduced with permission from author P. Wawryka [12].

The first (1) electrode Figure 4 was carefully placed over the BR muscle (m. brachioradialis) Figure 1. The remaining electrodes (2–8) were placed on a prototype band, as shown in Figure 4 and Figure 5, using eight sensors arranged in a circle. Positioning of sensors on the forearm (over selected muscles) is shown in Figure 1.

The electrode placement was carefully selected, as the spatial configuration significantly affects both the accuracy of signal processing and the performance of machine learning (ML) algorithms and artificial neural networks (ANNs) used for hand gesture recognition.

Prior to each measurement session, reference noise levels were recorded. Following electrode attachment, a stabilization period of approximately 5–20 s was required to ensure optimal electrical contact with the skin. It should be noted that electromagnetic interference (EMI) from power supply wiring within walls may affect the quality of the recorded EMG signals (Table 1).

All sEMG sensors were placed in accordance with the SENIAM guidelines (Surface Electromyography for the Non-Invasive Assessment of Muscles) [1], ensuring standardization and reliability of the measurements.

**Figure 6:**
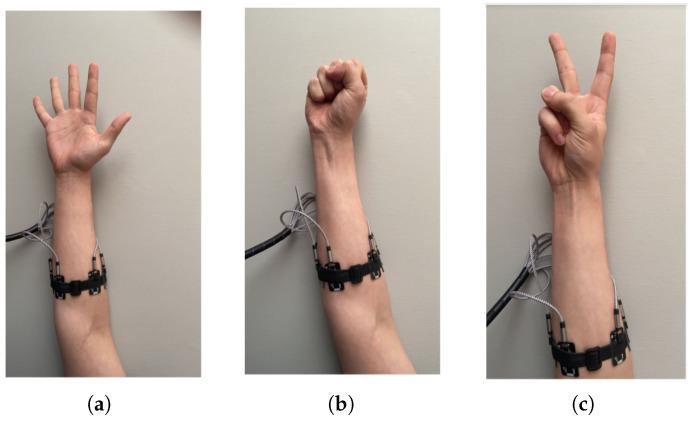

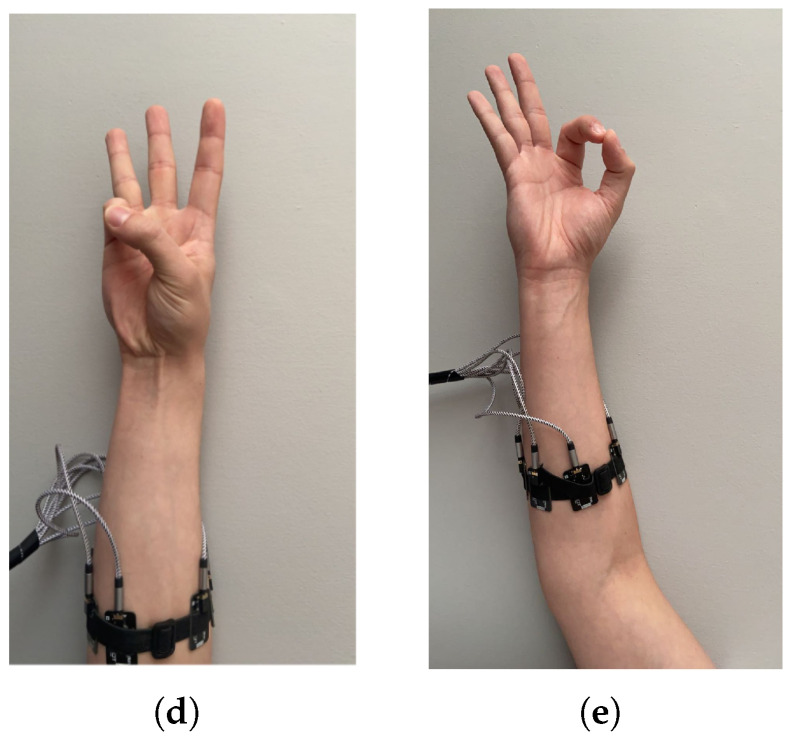
(**a**) Gesture straight fingers. (**b**) Gesture clenched hand. (**c**) Gesture victory. (**d**) Gesture middle fingers straight. (**e**) Gesture OK. Reproduced with permission from author P. Wawryka [12].

The following hand gestures were arbitrarily selected for analysis and utilized in the surface electromyography (sEMG) study:

1. Straight fingers—Figure 6a;
2. Clenched hand—Figure 6b;
3. Victory—Figure 6c;
4. The middle fingers straight—Figure 6d;
5. OK gesture—Figure 6e.

### 2.3. Software

A dedicated software application for data acquisition was developed using the C++ programming language within the Qt Creator 5.0.2 integrated development environment. This application enables real-time visualization of electromyographic (EMG) signals and their recording into a multi-channel WAVE file format [13].

The Hilbert–Huang Transform (HHT) [14,15] was applied in the MATLAB 2024b environment to extract selected features of the EMG signals [13]. Additional feature extraction procedures were also carried out using MATLAB 2024b software [16].

Machine learning models, including regularized logistic regression and artificial neural networks (ANNs), were implemented using JMP Student Edition 18.2.0 [17].

The study was conducted on a cohort of 109 healthy volunteers, with the objective of classifying four predefined gestures of the right hand. Electrode No. 1 was positioned on the brachioradialis (BR) muscle (Figure 1), with subsequent electrodes placed in sequence laterally outward from the participant’s perspective (Figure 4).

## 3. Processing EMG Signal Features

### 3.1. Feature Extraction

In the initial phase of the analysis, electromyographic signals acquired from the bionic hand were subjected to a feature extraction process. The purpose of this step was to identify salient signal characteristics that could be utilized as input features for subsequent processing and analysis.

Depending on the computational methodology, the extracted features can be categorized into three distinct groups: features obtained directly from the raw signal, features derived from the signal envelope, and features extracted through signal decomposition techniques (Table 2).

In this study, EMG signals were acquired using an eight-channel measurement system. Data acquisition was performed for 18 defined factors, each representing different states or tasks. Consequently, eight data channels were obtained for each of the 18 factors, resulting in a data set comprising 144 scalar features derived from unprocessed EMG signals.

Additionally, 16 additionalnal scalar features were incorporated into the set of features. These additional features represent the mean and standard deviation calculated from the upper-range envelope of the signal for each of the eight individual channels.

**Figure 7:**
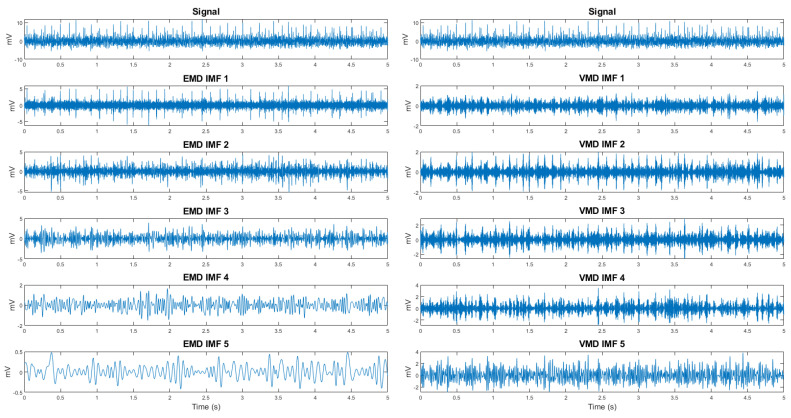
An example of the intrinsic mode functions (IMFs) and Variational Mode Decomposition (VMD) modes, derived from a single channel of surface electromyography (sEMG), illustrating the decomposition of a 5−s gesture.

An additional set of features was derived from the decomposition (Figure 7) of the signal using Empirical Mode Decomposition (EMD) [18] and Variational Mode Decomposition (VMD) [19]. From the EMD decomposition, the seven fundamental intrinsic mode functions (IMFs) were selected, while five modes were chosen from the VMD decomposition for each of the eight electromyographic (EMG) signal channels. This resulted in a total of 96 components. Subsequently, the energy and approximate entropy were calculated for each of these components, resulting in a final set of 192 novel features.

The comprehensive feature set consisted of 352 distinct scalar features, encompassing a diverse range of information extracted from EMG signals. This set included 144 features derived from unprocessed EMG data, 16 features representing the mean and standard deviation of the upper-range signal envelope, and 192 features obtained through EMD and VMD analysis. These features collectively aimed to capture the multifaceted characteristics of muscle activity, providing a robust foundation for subsequent analytical and modeling procedures.

### 3.2. Data Preprocessing

The data set, containing 362 instances, was partitioned into training, validation, and test subsets with a distribution of 78% for training, 11% for validation, and 11% for testing. The subsets were randomly selected, maintaining the representation of all five gesture classes within each subset.

Due to the significant class imbalance observed within the gesture dataset, the Receiver Operating Characteristic Area Under the Curve (ROC AUC) was used as a primary evaluation metric for predictive models. This metric was selected to provide a robust assessment of model performance, where it is particularly useful in scenarios where traditional accuracy measures may be misleading due to uneven class distributions. The ROC AUC assesses the model’s ability to discriminate between classes across various threshold settings.

### 3.3. Building Models

Research has consistently shown that advanced machine learning algorithms can achieve high accuracy in classifying electromyographic (EMG) signals for the identification of specific hand movements [20]. Building on this, investigations were conducted to evaluate various machine learning models, specifically emphasizing regularized and dimensionality-reduced logistic regression models, alongside neural network classifiers. While the classification performance of the selected methods was largely comparable, neural network classifiers demonstrated a marginal superiority. Nevertheless, logistic regression models present distinct advantages, including notable resistance to overfitting, simplicity of implementation, computational efficiency, and enhanced interpretability. Collectively, these attributes render logistic regression a highly appropriate solution for controlling bionic prostheses.

Among the logistic regression models, regularized variants employing L1 (LASSO—Least Absolute Shrinkage and Selection Operator [21]), Ridge Regression (L2) [22] and Elastic Net [23] penalties were evaluated. These regularization techniques were implemented to mitigate the risk of overfitting, particularly in scenarios with a high number of input features relative to the sample size. By adding a penalty term to the loss function, these methods constrain the magnitude of the coefficient estimates. This helps in reducing the impact of multicollinearity.

The LASSO method modifies the standard cost function (typically the sum of squared residuals for linear regression or negative log-likelihood for logistic regression) by adding an L1 penalty (L1 norm) to the sum of the absolute values of the regression coefficients.

(1) $$\min_{\beta_{0},\beta }\left\{\frac{1}{2n}\sum_{i=1}^{n}{\left(y_{i}-\left(\beta_{0}+x_{i}^{T}\beta \right)\right)}^{2}+\lambda \sum_{j=1}^{p}\left|\beta_{j}\right|\right\},$$

where

- $y_{i}$ is the observed value of the dependent variable for the *i*-th observation;
- $x_{i}$ is the vector of independent variables for the *i*-th observation;
- $\beta_{0}$ is the intercept term;
- β is the vector of regression coefficients for the predictors;
- *n* is the number of observations;
- *p* is the number of predictors;
- λ (lambda) is the regularization parameter (penalty strength), controlling the degree of coefficient shrinkage, where a larger λ indicates a stronger penalty;
- $\sum_{j=1}^{p}\left|\beta_{j}\right|$ is the L1 penalty, representing the sum of the absolute values of the coefficients.

This formula can be simplified to minimize a cost function (also known as a loss function). This cost function combines the prediction error with a regularization term as defined below:

(2) $${Cost}_{LASSO}=Prediction\;Error+\lambda \sum_{j=1}^{p}\left|\beta_{j}\right|$$

For the LASSO regularization, a penalty fraction (λ) of 0.001 was applied.

Elastic Net combines the L1 penalty of LASSO with an L2 penalty (sum of squared coefficients, $\sum \beta_{j}^{2}$), which is characteristic of Ridge Regression. Elastic Net offers a balance between feature selection and coefficient shrinkage, potentially outperforming either L1 or L2 alone in situations with groups of correlated variables.

(3) $$\min_{\beta_{0},\beta }\left\{\frac{1}{2n}\sum_{i=1}^{n}{\left(y_{i}-\left(\beta_{0}+x_{i}^{T}\beta \right)\right)}^{2}+\lambda \left(\alpha \sum_{j=1}^{p}\left|\beta_{j}\right|+\frac{\left(1-\alpha \right)}{2}\sum_{j=1}^{p}\beta_{j}^{2}\right)\right\},$$

where

- $y_{i}$, $x_{i}$, $\beta_{0}$, β, *n*, and *p* have the same meaning as in LASSO;
- λ (lambda) is the regularization parameter, controlling the overall penalty strength;
- α (alpha) is the mixing parameter, in the range of [0,1];
- If α=1, Elastic Net becomes LASSO (L1 penalty only);
- If α=0, Elastic Net becomes Ridge (L2 penalty only);
- For 0<α<1, Elastic Net is a combination of both penalties;
- $\sum_{j=1}^{p}\left|\beta_{j}\right|$ is the L1 penalty;
- $\sum_{j=1}^{p}\beta_{j}^{2}$ is the L2 penalty (sum of squared coefficients).

Consistent with other regularization techniques, Elastic Net regularization also seeks to minimize a cost function, whose formulation can likewise be simplified as follows:

(4) $${Cost}_{ElasticNet}=Prediction\;Error+\lambda \left(\alpha \sum_{j=1}^{p}\left|\beta_{j}\right|+\frac{\left(1-\alpha \right)}{2}\sum_{j=1}^{p}\beta_{j}^{2}\right)$$

Elastic Net regularization was performed with an α value of 0.99 and a penalty fraction (λ) of 0.001.

The performance of these regularized logistic regression models was compared to assess their suitability for the specific classification task, considering factors such as predictive accuracy, model complexity, and interpretability.

In addition to the regularization methods, a feature selection technique employing Recursive Feature Elimination (RFE [24]) was also implemented. RFE is a wrapper-type feature selection algorithm that operates by iteratively fitting the model and removing the least important features based on the model’s coefficient magnitudes (in the case of linear models like logistic regression). This process is repeated until the desired number of features is reached. The rationale behind employing RFE was to identify the most informative subset of features for the classification task, potentially improving model parsimony, reducing computational cost, and enhancing generalization performance by removing noisy or redundant variables. A modification to the RFE method was introduced, wherein the selected feature subset maximized the minimum F1-score obtained for any category. Specifically, the objective was to find the subset of features that maximized the F1 score as follows:

(5) $$\max_{\mathrm{feature}\mathrm{subset}}\left(\min_{c\in \mathrm{categories}}F1_{c}\right)$$

where F1c represents the F1-score for category c.

**Figure 8:**
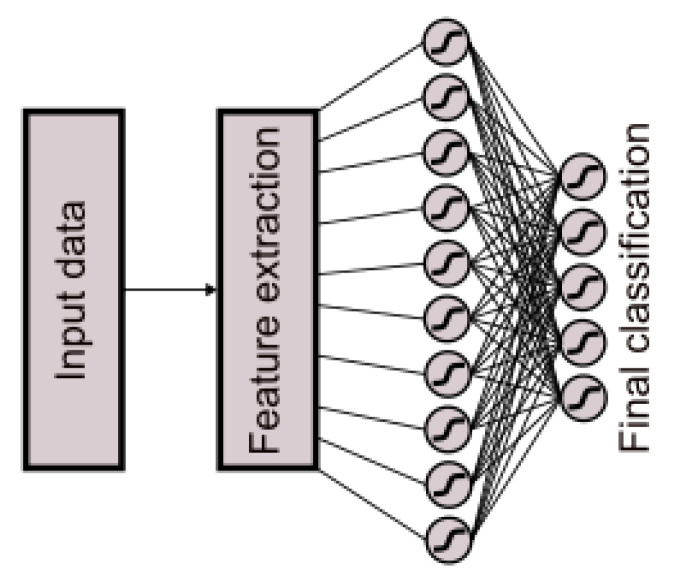
Neural network classification: data preprocessing and transformation pipeline.

Within the experimental framework, neural network classifier models, in addition to regression models, exhibited significant performance. Therefore, a neural network classifier model was implemented, comprising 10 neurons employing the tanh activation function in the hidden layer and 5 neurons using the tanh activation function in the output layer (Figure 8).

In JMP, hyperparameters were primarily determined through internal validation mechanisms within the platform, such as cross-validation or validation set performance, which JMP automatically optimizes. This approach allows the software to iteratively search for and select the parameter values that yield the best model performance based on the chosen validation metric. JMP, like many advanced statistical and analytical software packages, typically does not disclose the specific fixed name of the optimization algorithm used “under the hood” for its artificial neural networks (ANN). Instead, it focuses on delivering optimized results and offers the user certain configuration options that influence the optimization process.

Although direct access to neural network hyperparameters was unavailable, a significant benefit emerged from the unified testing environment provided by JMP. This enabled a more robust and comparable evaluation of the machine learning algorithms.

Furthermore, an exploration of signal classification capabilities was conducted employing GRU and LSTM architectures applied to unprocessed signal data. However, the insufficient sample size within the data set limited the ability to establish the effectiveness of these network models.

## 4. Implementation Simple Bionic Hand

### Model of Bionic Hand—Hardware

**Figure 9:**
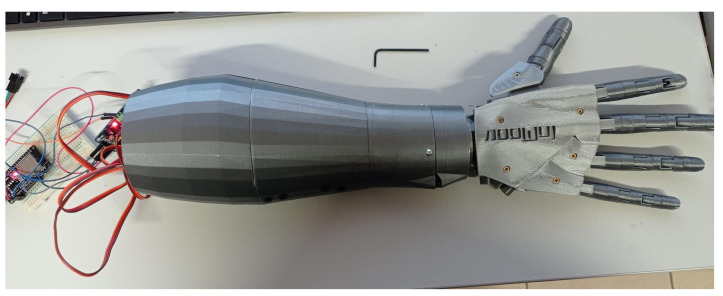
Inner side of 3D printed bionic hand.

**Figure 10:**
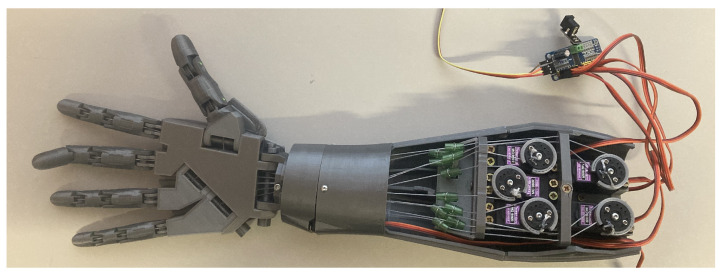
The outside of the 3D printed bionic hand.

The bionic hand model (Figure 9 and Figure 10) was made using a 3D printer (Prusa i3 MK3S+, Prague, Czech Republic) equipped with the Multi Material 2 upgrade. All components of the model were designed by Gael Langevin [25], who made them publicly available on the Thingiverse platform under a Creative Commons license.

Individual parts of the prototype were printed using Spectrum PETG filament (1.75 mm diameter, Silver Star color). This material was selected due to its favorable mechanical properties, including high durability, flexibility, and resistance to moisture-induced degradation. The assembled components were joined using appropriate fasteners and adhesive to ensure structural integrity.

The bionic hand is actuated by six Tower Pro MG996R servo motors, each with an angular range of 180°, and controlled via a PCA9685 PWM driver board (Adafruit 815) connected to the main controller. The first servo is responsible for wrist articulation, while the remaining five control the individual fingers by tensioning or releasing braided fishing lines.

To ensure reliable actuation and minimize elastic deformation, high-strength braided fishing line (PowerPro Spectra, 0.76 mm diameter, tensile strength 90 kg) was used. Each line was routed through dedicated guide holes to prevent tangling. In order to maintain consistent tension in the tendons of each finger, return springs were incorporated into the tensioning mechanism.

The servo control system was integrated with the same LPC1347 microcontroller board that was used during the initial stages for surface electromyography (sEMG) signal acquisition, enabling a compact and unified hardware architecture.

Each servo motor was individually calibrated to define precise initial and terminal positions for each finger. Finger flexion is achieved by varying the servo positions, resulting in proportional joint articulation. When all servo motors are set to their zero-angle positions, the hand assumes a fully open posture, which serves as the system’s reference configuration.

## 5. Results and Discussion

The experiment assessed the effectiveness of recognizing five distinct hand gestures using both regularized logistic regression models and artificial neural networks (ANNs). These two methods demonstrated superior classification performance compared to other initially evaluated algorithms, including k-Nearest Neighbors (k-NN), Support Vector Machines (SVMs), Random Forests, and Decision Trees.

The analysis was based on complete 5-second signal sequences, from which scalar features were subsequently extracted. The use of full-length sequences allowed for improved model fitting to representative patterns in the EMG signals, including potential artifacts that could arise during the real-world operation of the bionic hand.

The results indicate that both artificial neural networks (ANNs) employing sigmoidal activation functions and regularized logistic regression models achieved comparable performance (see Table 3 and Table 4). Neural networks exhibited a slightly greater tendency to overfit the training data. Additionally, the ANNs utilized the full set of available features, whereas the regularized models selected a subset—103 features in the case of LASSO regression and 135 features with Elastic Net regularization.

Overall, the most favorable results were achieved through the application of logistic regression with Elastic Net regularization. Considering the lowest classification accuracies across gestures, LASSO and Elastic Net yielded the highest values. It is also pertinent to note that the performance of the ANNs varied depending on the local minimum of the loss function identified by the optimization algorithm. The table presents the optimal outcome attained across multiple experimental runs, which consequently positions the results obtained for the logistic regression methods in a more advantageous perspective.

**Figure 11:**
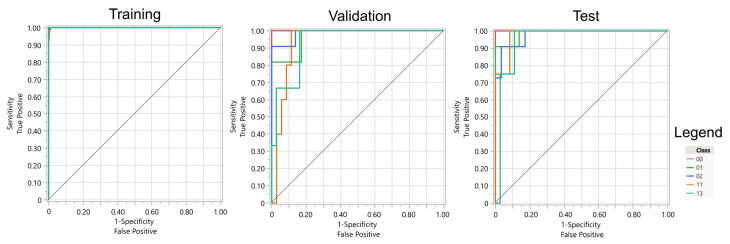
ROC curves for ANN performance on training, validation, and test sets.

**Figure 12:**
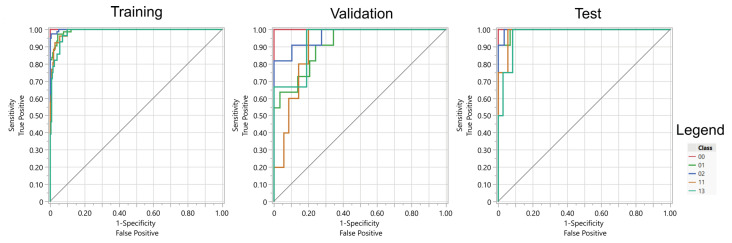
ROC curves for elastic performance on training, validation, and test sets.

The absence of a significant advantage of ANNs over regression methods on the test data suggests that Elastic Net or LASSO are the recommended models for practical application (see Figure 11 and Figure 12). Furthermore, the high generalization capabilities inherent in regularized regression models, stemming from their propensity to avoid overfitting the data, further support their adoption. Moreover, regression models offer transparency, ease of interpretation, and straightforward implementation on the bionic hand’s control system. These models also possess a significantly smaller computational footprint, thereby enabling a more rapid response of the bionic hand to EMG signals.

A primary limitation of employing neural networks in this study lies in their substantial demand for diverse and extensive datasets, the acquisition of which necessitates considerable time and financial resources without providing an absolute guarantee of successful implementation.

The ElasticNet model, as demonstrated in the provided example, incorporated nearly all features enumerated in Table 1 during its construction. Notably, the features Kurtosis, Approximate Entropy, and Energy were entirely excluded from the model training process. All other features participated to a greater or lesser extent in the training process of the ElasticNet model.

This study employed numerous features for constructing the Elastic Net regression model, from which several statistically significant ones were identified. The assessment of statistical significance was based on rejecting the null hypothesis of a zero regression coefficient (with a *p*-value below 0.05).

The most significant predictors, exhibiting non-zero coefficients in the model, include the following:

- Signal skewness in channel 3.
- Mean signal value in channel 1.
- Entropy of the first intrinsic mode function (IMF) from VMD decomposition in channel 8.
- Mean signal value in channel 4.
- Mean signal frequency in channel 6.
- Energy of the fifth IMF from EMD decomposition in channel 4.
- Correlation Dimension in channel 3.
- Total Harmonic Distortion (THD) in channel 6.

These findings indicate that the most influential factors in the model were derived from data originating in channels 1, 3, 4, 6, and 8 (Figure 1). A detailed description of all utilized features is beyond the scope of this section. The diverse nature of the identified features—ranging from statistical moments (skewness, mean) to spectral (frequency, THD) and decomposition-based (IMF entropy, energy) as well as non-linear dynamics (Correlation Dimension) metrics—underscores the complexity of the underlying process and the necessity of a multi-faceted approach to feature extraction.

In the LASSO regression model, twelve predictors demonstrated statistical significance, with their *p*-values falling below the 0.05 threshold. The list of these significant predictors is as follows (those also identified in the Elastic Net model are italicized):

- Mean upper envelope of the signal in channel 5.
- Correlation Dimension in channel 2.
- Signal skewness in channel 3.
- Entropy of the first intrinsic mode function (IMF) from VMD decomposition in channel 8.
- Mean signal value in channel 1.
- Mean signal frequency in channel 6.
- Shape Factor in channel 1.
- Correlation Dimension in channel 3.
- Mean signal value in channel 4.
- Energy of the fifth IMF from EMD decomposition in channel 4.
- Energy of the first IMF from VMD decomposition in channel 6.
- Total Harmonic Distortion (THD) in channel 6.

The identification of these statistically significant predictors in the LASSO model highlights the most influential features contributing to the model’s predictive power. The substantial overlap with features selected by the Elastic Net model (8 out of 12) suggests a robust set of key indicators across both regularization techniques. This consistency underscores the reliability of these particular features in capturing the underlying patterns within the data. Furthermore, the inclusion of additional unique features by the LASSO model, such as the mean upper envelope in channel 5 and the form factor in channel 1, indicates that while core predictive elements are shared, each regularization method may prioritize slightly different aspects of the data—potentially due to their distinct penalty functions (L1 for LASSO’s sparsity vs. combined L1/L2 for Elastic Net’s grouping effect).

## 6. Conclusions

In summary, although artificial neural networks (ANNs) exhibited comparable or, in certain cases, marginally superior performance in gesture classification, several considerations advocate for the use of regularized logistic regression models—specifically Elastic Net and LASSO—in the practical implementation of bionic hand control based on EMG signals. The absence of a definitive advantage of ANNs on the test dataset, combined with their intrinsic dependence on large and diverse training datasets—whose acquisition is both resource-intensive and does not ensure optimal performance—constitutes a significant practical limitation.

Conversely, regularized regression models constitute a compelling alternative due to their superior generalization capabilities, which reduce the risk of overfitting—an issue more prominently observed in the evaluated artificial neural networks. Moreover, the intrinsic transparency, interpretability, and ease of implementation of these models within the bionic hand control system represent significant advantages. Their lower computational demands enable more rapid processing, thereby facilitating a potentially critical real-time response of the prosthetic limb to the user’s intended gestures—an essential requirement for practical applications.

Conclusions drawn from a review of advancements in robotic upper-limb prostheses [26] indicate that a major limitation of machine learning methods is their black-box nature coupled with high computational demands. The application of effective models based on regularized logistic regression addresses these fundamental challenges inherent to conventional ML approaches.

Considering these factors—particularly the trade-offs between performance, data requirements, interpretability, implementability, and real-time responsiveness—regularized logistic regression methods, notably Elastic Net and LASSO, emerge as a more pragmatic and potentially efficacious choice for controlling bionic hands in this context.

The classification accuracy of gestures was substantially improved compared to the initial study [11], owing to an expanded feature set employed to characterize EMG signals. An additional advantage of this increased feature dimensionality was the feasibility of applying efficient logistic regression techniques that demonstrated competitive performance relative to artificial neural networks (ANNs). Furthermore, the evaluation methodology for gesture classification was revised from accuracy-based metrics [11] to the Receiver Operating Characteristic (ROC) curve and Area Under the Curve (AUC). This shift to more robust model quality indicators provides a better representation of classification performance, especially when addressing imbalanced datasets.

A detailed analysis of feature extraction methods contributing to the development of controllable prosthetic hands has been conducted [27]. Beyond applications in computer or mobile device gesture control, research in this domain holds potential for prostheses addressing hand hemimelia. Such prosthetic systems could be further enhanced by incorporating modules supported by Inertial Measurement Units (IMUs) to detect hand rotation.

All experiments were performed using MATLAB 2024b [16], JMP Student Edition 18.2.0 [17], and Python 3.13 software, with JMP Student Edition 18.2.0 additionally employed for ROC AUC visualization.

## Figures and Tables

**Table 1:** Parameters of the sEMG signal acquisition module.

Settings	Values
Number of sEMG channels (sensors)	8
Analyzed frequency range	1–1024 Hz
Signal amplification	1000
Sampling frequency	2048 Hz
Signal acquisition time	5 s
Smooth signal	5 samples

**Table 2:** EMG signal features for bionic hand movement classification.

Unprocessed signal	1. Mean, 2. RMS, 3. Shape Factor, 4. SNR, 5. THD, 6. SINAD, 7. Peak Value, 8. Crest Factor, 9. Clearance Factor, 10. Impulse Factor, 11. Kurtosis, 12. Skewness, 13. Approximate Entropy, 14. Energy, 15. Mean Frequency, 16. Mean Peaks, 17. Std Peaks, 18. Correlation Dimension
Envelopes	1. Mean of upper envelope, 2. Std of upper envelope
Decomposition	1. Entropy of imf, 2. Enegry of imf

**Table 3:** Gesture classification performance (regularized logistic regression).

	Ridge Regression			Lasso Regression			Elastic Net Regression		
Gesture	Train	Val	Test	Train	Val	Test	Train	Val	Test
1	1.0000	1.0000	1.0000	1.0000	1.0000	1.0000	1.0000	1.0000	1.0000
2	0.9930	0.9028	1.0000	0.9914	0.9060	0.9875	0.9930	0.9122	0.9937
3	0.9983	0.9561	0.9969	0.9990	0.9592	0.9937	0.9987	0.9655	0.9969
4	0.9946	0.9143	0.9653	0.9874	0.8857	0.9792	0.9896	0.9029	0.9861
5	0.9873	0.9369	0.9375	0.9821	0.9369	0.9722	0.9845	0.9369	0.9722

**Table 4:** Gesture classification performance (RFE and ANNs).

	RFE Regression			ANN (Linear Function)			ANN (Tanh Function)		
Gesture	Train	Val	Test	Train	Val	Test	Train	Val	Test
1	1.0000	1.0000	1.0000	1.0000	1.0000	0.9967	1.0000	1.0000	1.0000
2	1.0000	0.7962	0.9263	0.9360	0.9655	0.9185	0.9998	0.9687	0.9875
3	1.0000	0.7241	0.8777	0.9871	0.9655	0.9028	0.9999	0.9875	0.9781
4	1.0000	0.8443	0.7917	0.9593	0.9600	0.9097	1.0000	0.9371	0.9792
5	1.0000	0.9369	0.8819	0.9100	0.9459	0.9236	0.9997	0.9369	0.9514

## Data Availability

The datasets used and analysed during the current study available from the corresponding author on reasonable request.
